# Supplementary material for: A Highly Sensitive and Selective Nano-Fluorescent Probe for Ratiometric and Visual Detection of Oxytetracycline Benefiting from Dual Roles of Nitrogen-Doped Carbon Dots
Source: Nanomaterials (Basel). 2022 Dec 4;12(23):4306. doi: 10.3390/nano12234306 (PMC9735690; doi:10.3390/nano12234306)
Supplement: Supplementary file 1 [file nanomaterials-12-04306-s001.zip › nanomaterials-2046152-supplementary.pdf]

Supplementary Materials

A Highly Sensitive and Selective Nano-Fluorescent Probe for Ratiometric and Visual Detection of Oxytetracycline Benefiting from Dual Roles of Nitrogen-Doped Carbon Dots

Huifang Wu *, Mengqi Xu, Yubing Chen, Haoliang Zhang, Yongjun Shen and Yanfeng Tang *

Nantong Key Lab of Intelligent and New Energy Materials, School of Chemistry and Chemical Engineering, Nantong University, Nantong 226019, China

* Correspondence: whf0125@ntu.edu.cn (H.W.); tangyf@ntu.edu.cn (Y.T.)

Apparatus and characterization

The morphologies and sizes of the NCDs were characterized by JEM-2100F transmission electron microscopy (TEM, JEOL, Japan). The elemental compositions and contents of NCDs were analyzed with SIRION-100 X-ray energy dispersive spectroscopy (EDX, PEI, Netherlands). The X-ray diffraction (XRD) pattern was recorded on a D8 advance X-ray diffractometer (Bruker, Germany). Chemical compositions and valency were analyzed by X-ray photoelectron spectroscopy (XPS) performed on a Thermo Kalpha spectrometer (Thermo Fisher Scientific, USA) using a 1486.6 eV Al Kα line excitation source. Fourier-transform infrared (FT-IR) spectra were recorded on a Nicolet IS10 FT-IR spectrometer (Thermo Fisher Scientific, USA) with KBr plates. Fluorescence spectra were collected with an F-7000 spectrofluorimeter (Hitachi, Japan) with the photomultiplier tube voltage set at 400 V. Fluorescence lifetime curves and fluorescence quantum yield were generated on a FLS-1000 steady-state and time-resolved spectrofluorimeter (Edinburgh, England). UV-Vis spectra were recorded on a UV-3600 spectrophotometer (Shimadzu, Japan). All pH measurements were performed on a FE28-standard pH meter (Mettler Toledo, Switzerland).

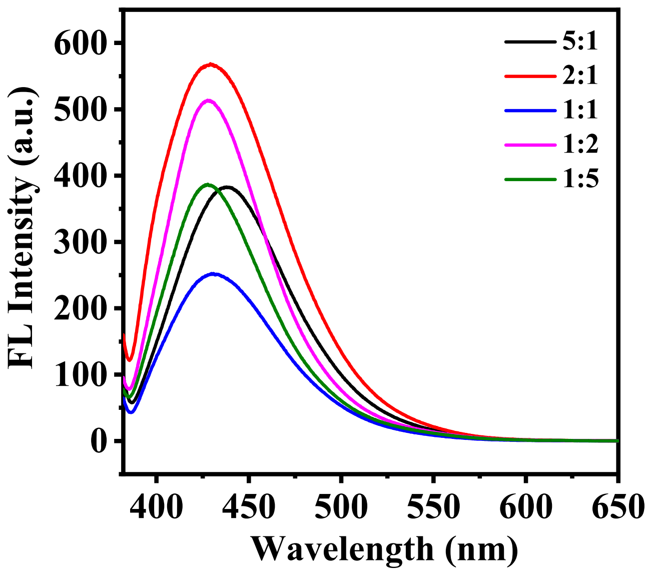

Figure S1. Fluorescence spectra of NCDs synthesized with different M_{CA}/M_{UA}.

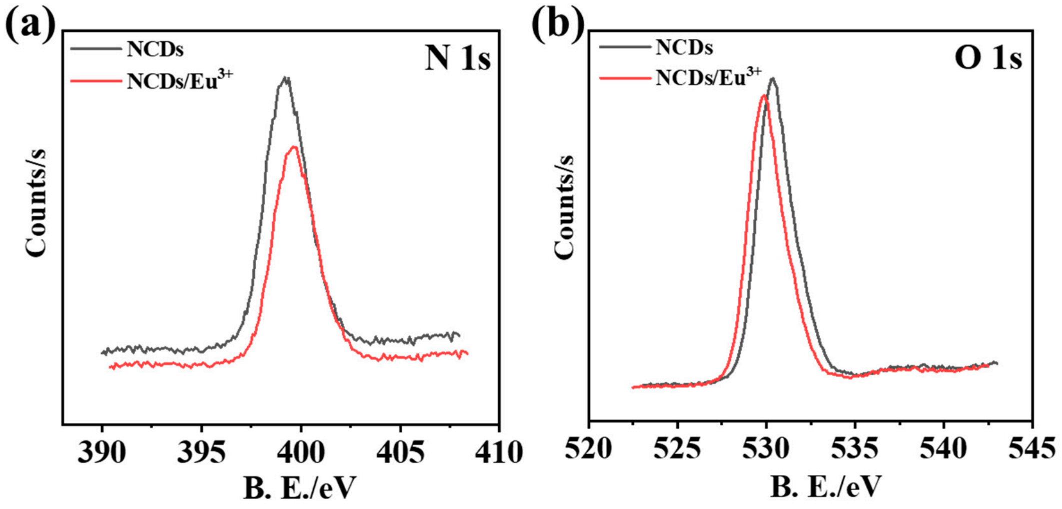

Figure S2. The high-resolution N1s (a) and O1s (b) spectra of NCDs and NCDs/Eu^{3+}.

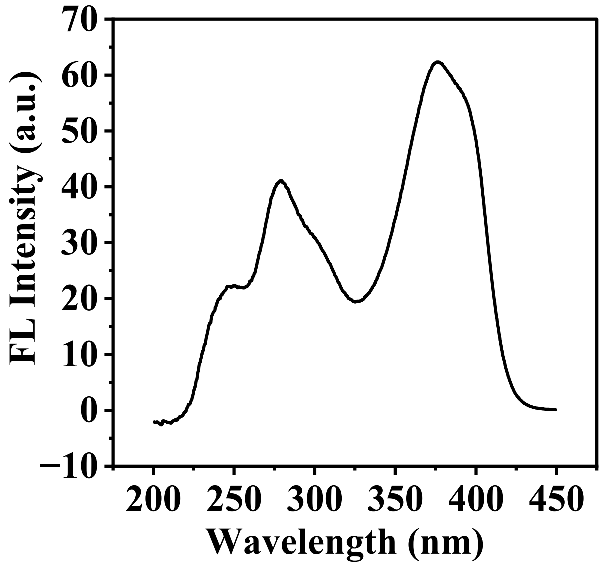

Figure S3. Excitation spectra of NCDs/Eu^{3+}–OTC.

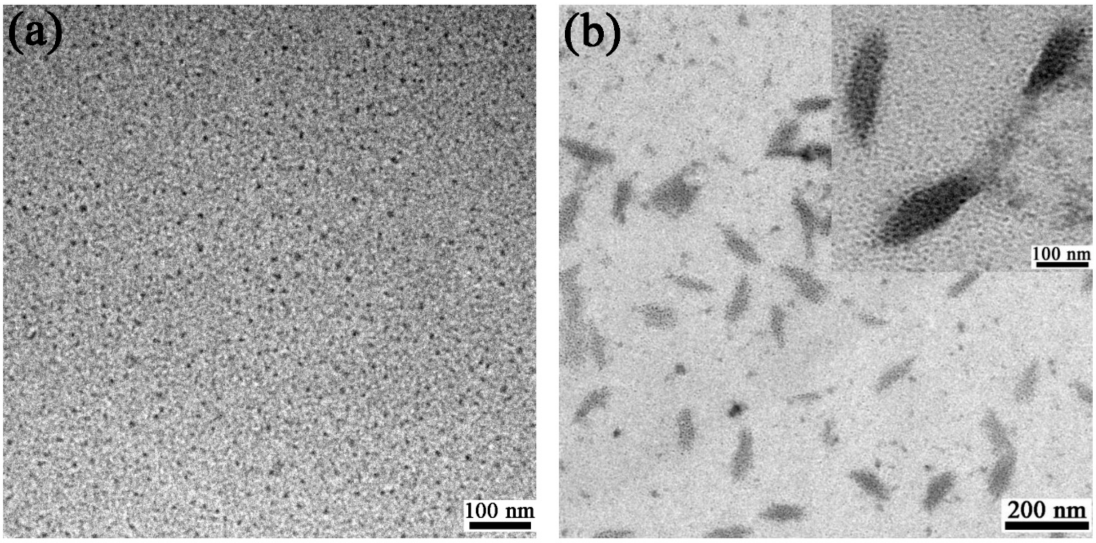

Figure S4. TEM images of NCDs/Eu^{3+} before (a) and after (b) incubation with OTC.

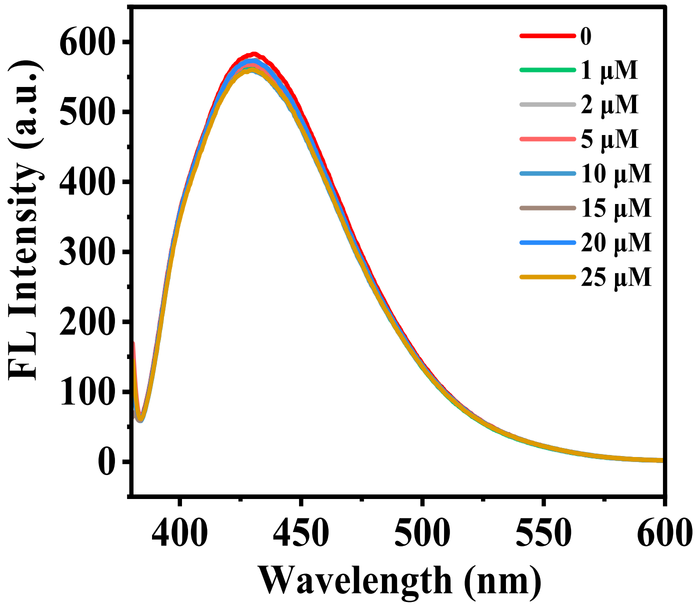

Figure S5. Effect of OTC at different concentrations on the fluorescence spectra of NCDs.

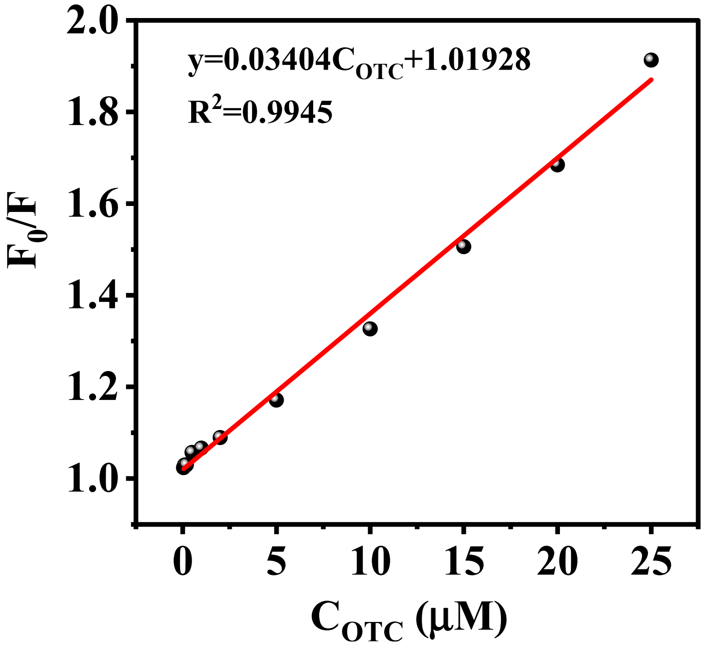

Figure S6. Stern–Volmer plot of NCDs/Eu^{3+} probe after addition of OTC in the range of 0.1–25 μM.

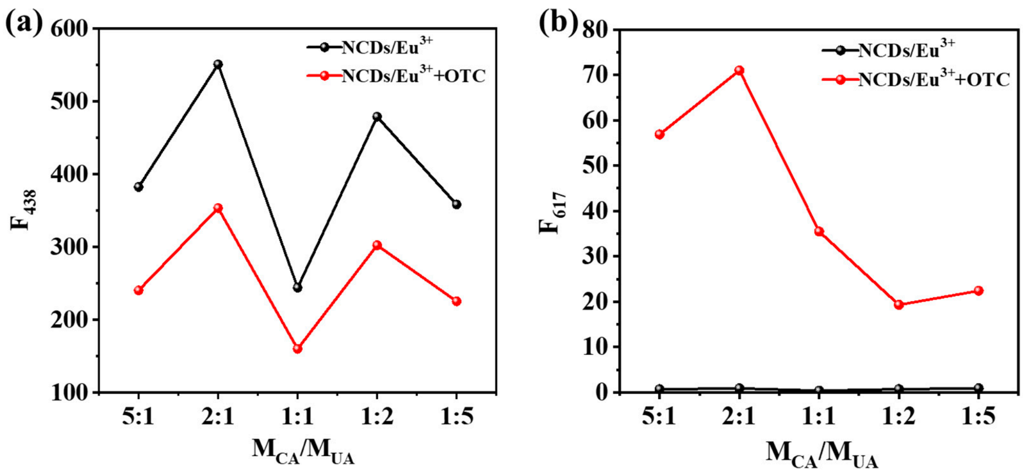

Figure S7. Effect of M_{CA}/M_{UA} on F_{438} and F_{617} of the probe before (a) and after (b) incubation with OTC. NCDs: 20 mg·L^{-1}; Eu^{3+}: 60 μM; OTC: 20 μM; HEPES buffer: 10 mM (pH=7.0).

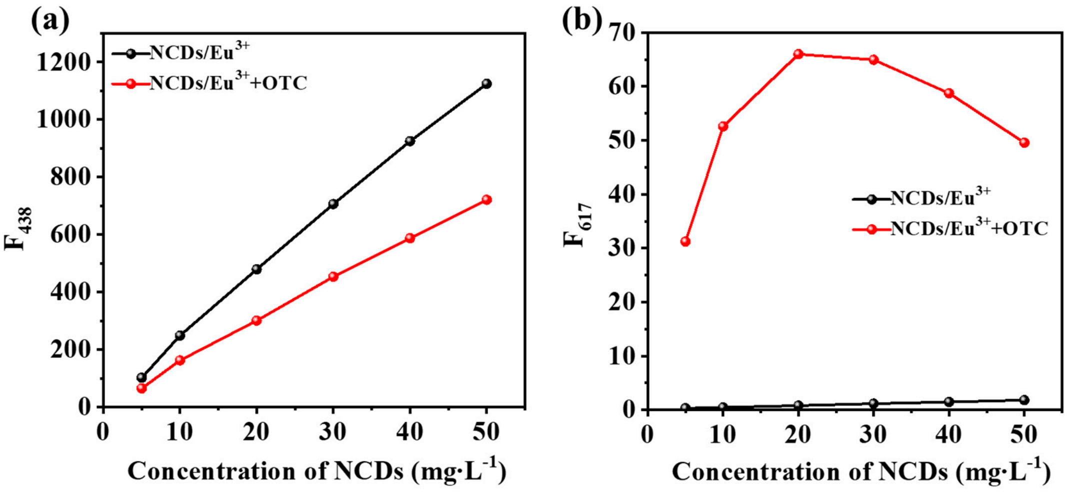

Figure S8. Effect of NCD concentration on F_{438} and F_{617} of the probe before (a) and after (b) incubation with OTC. Eu^{3+}: 60 μM; OTC: 20 μM; HEPES buffer: 10 mM (pH=7.0).

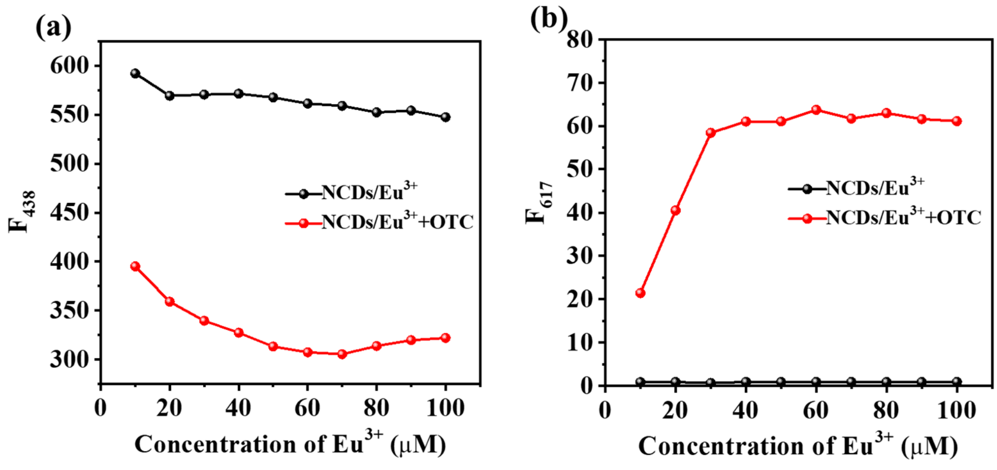

Figure S9. Effect of Eu^{3+} concentration on F_{438} and F_{617} of the probe before (a) and after (b) incubation with OTC. NCDs: 20 mg·L^{-1}; OTC: 20 μM; HEPES buffer: 10 mM (pH=7.0).

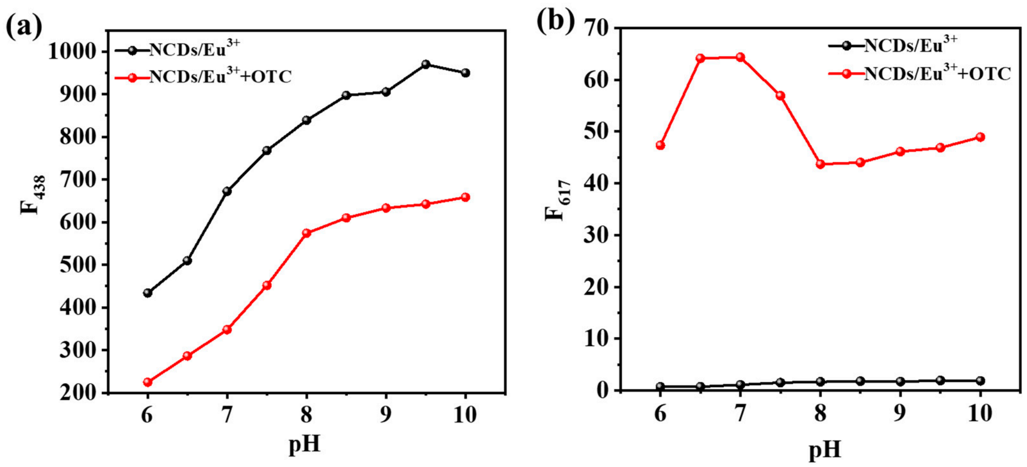

Figure S10. Effect of pH on F_{438} and F_{617} of the probe before (a) and after (b) incubation with OTC. Eu^{3+}: 60 μM; OTC: 20 μM; HEPES buffer: 10 mM (pH=7.0).

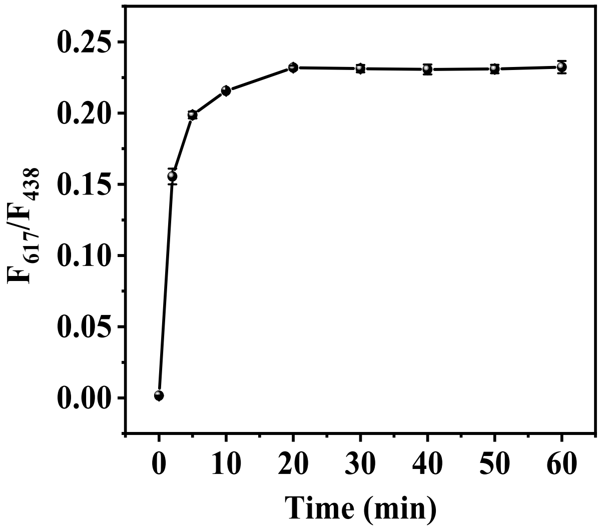

Figure S11. Effect of incubation time on F_{617}/F_{438}. NCDs: 20 mg·L^{-1}; Eu^{3+}: 60 μM; OTC: 20 μM; HEPES buffer: 10 mM (pH=7.0).

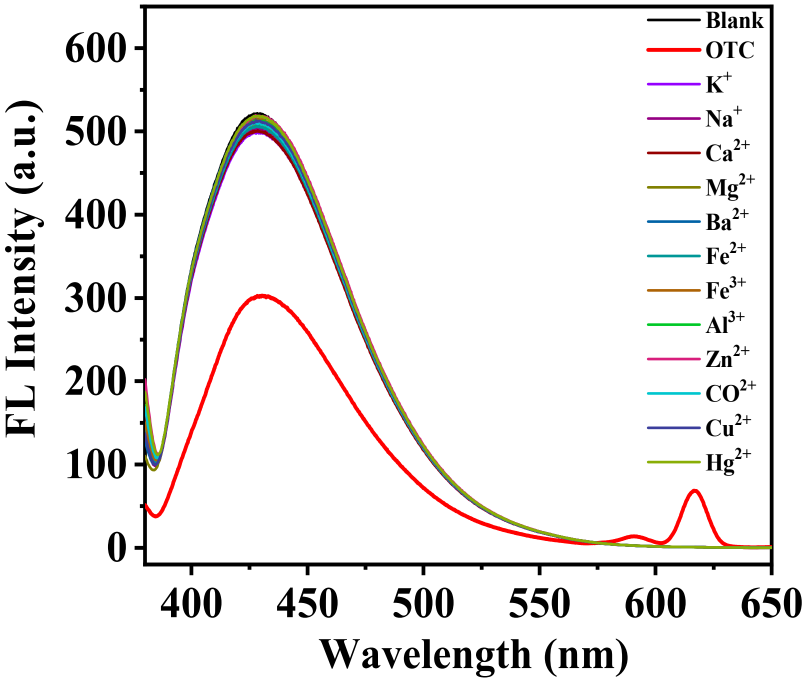

Figure S12. The effect of other cations on the fluorescence spectra of NCDs/Eu^{3+}.

Table S1. The reproducibility of NCD physical properties.

NCDs	Particle size (nm)	Fluorescence quantum yield (%)	Fluorescence lifetime (ns)	Excitation wavelength- and pH-dependent optical property
NCDs 1	4.89 ± 0.37	16.10	4.01	yes
NCDs 2	4.02 ± 0.30	14.95	3.03	yes
NCDs 3	4.55 ± 0.25	15.48	3.17	yes

Table S2. The reproducibility of NCDs analytical properties for OTC detection.

NCDs	Quantitative range for OTC	LOD for OTC	Selectivity for OTC
NCDs 1	0.1–25 μM	40 nM	yes
NCDs 2	0.1–25 μM	18 nM	yes
NCDs 3	0.1–25 μM	29 nM	yes

Table S3. Comparison of fluorescent probes for OTC detection.

Material applied	Targets	Linear range	LOD	Ref.
piperazine modified CQDs	OTC	0–10 μM	–	[1]
GQDs-Eu^{3+}	TC[a]/OTC/DXC[b]	0–20 μM	8.2 nM	[2]
Al-MOF	TC[a]/OTC/DXC[b]	0–72.33/0–86.67/0–66.67 μM	26/62/40 nM	[3]
luminescent MOF (In-sbdc)	TC[a]/OTC/CTC[c]	0–30 μM	0.28/0.30/0.30 μM	[4]
BNQD-Eu^{3+}	TC[a]/OTC/DXC[b]	0–50 μM	19/104/28 nM	[5]
CDs–Eu^{3+}	OTC	2.5–10 μM	9.59 nM	[6]
DPA-Ce-GMP-Eu	OTC	0.01–45 μM	6.6 nM	[7]
CDs	OTC	0–40 μM	410 nM	[8]

NCDs/Eu^{3+}	OTC	0.1–25 μM	29 nM	This work

[a] tetracycline;
[b] doxycycline;
[c] chlortetracycline;

References

[1] Yang L.; Zhao H.T.; Liu N.; Wang W. A target analyte induced fluorescence band shift of piperazine modified carbon quantum dots: a specific visual detection method for oxytetracycline. *Chem. Commun.* **2019**, *55*, 12364.

[2] Li W.; Zhu J.; Xie G.; Ren Y.; Zheng Y. Q. Ratiometric system based on graphene quantum dots and Eu3þ for selective detection of tetracyclines, *Anal. Chim. Acta.* **2018**, *1022*, 131-137.

[3] Li C.; Zhu L.; Yang W.; He X.; Zhao S.; Zhang X.; Tang W.; Wang J.; Yue T.; Li Z. Amino-functionalized Al-MOF for fluorescent detection of tetracyclines in milk. *J. Agric. Food Chem.*, **2019**, *67*, 1277-1283.

[4] Liu Q.; Ning D.; Li W. J.; Du X. M.; Wang Q.; Li, Y.; Ruan W. J. Metal-organic framework-based fluorescent sensing of tetracycline-type antibiotics applicable to environmental and food analysis. *Analyst*, **2019**, *144*, 1916-1922.

[5] Yang K.; Jia P.; Hou J.; Bu T.; Sun X.; Liu Y.; Wang L. Innovative dual-emitting ratiometric fluorescence sensor for tetracyclines detection based on boron nitride quantum dots and europium ions, *ACS Sustain. Chem. Eng.* **2020**, *8*, 17185-17193.

[6] Wang Q.; Li X.C.; Yang K.; Zhao S.J.; Zhu S.H.; Wang B.H.; Yi J.N.; Zhang Y.; Song X.Z.; Lan M.H. Carbon dots and Eu^{3+} hybrid-based ratiometric fluorescent probe for oxytetracycline detection. *Ind. Eng. Chem. Res.* **2022**, *61*, 5825−5832

[7] Wang T.; Mei Q.; Tao Z.; Wu H., Zhao M., Wang S.; Liu Y. A smartphone-integrated ratiometric fluorescence sensing platform for visual and quantitative point-of-care testing of tetracycline, *Biosens. Bioelectron.* **2020**, *148*, 111791-111798.

[8] Fu Y.Z.; Huang L.; Zhao S.J.; Xing X.J.; Lan M.H.; Song X.Z. A carbon dot-based fluorometric probe for oxytetracycline detection utilizing a Förster resonance energy transfer mechanism, *Spectrochim. Acta A* **2021**, *246*, 118947.
